# Supplementary material for: Chemotherapy and Radiotherapy Long-Term Adverse Effects on Oral Health of Childhood Cancer Survivors: A Systematic Review and Meta-Analysis
Source: Cancers (Basel). 2023 Dec 25;16(1):110. doi: 10.3390/cancers16010110 (PMC10777916; doi:10.3390/cancers16010110)
Supplement: Supplementary file 1 [file cancers-16-00110-s001.zip › cancers-2776218-supplementary.pdf]

Table S1. PRISMA 2020 Main Checklist.

Topic	No.	Item	Location where item is reported
**TITLE**			
**Title**	1	Identify the report as a systematic review.	Title
**ABSTRACT**			
**Abstract**	2	See the PRISMA 2020 for Abstracts checklist	
**INTRODUCTION**			
**Rationale**	3	Describe the rationale for the review in the context of existing knowledge.	Page 2
**Objectives**	4	Provide an explicit statement of the objective(s) or question(s) the review addresses.	Page 2
**METHODS**			
**Eligibility criteria**	5	Specify the inclusion and exclusion criteria for the review and how studies were grouped for the syntheses.	Page 3 - Focused question and eligibility criteria
**Information sources**	6	Specify all databases, registers, websites, organisations, reference lists and other sources searched or consulted to identify studies. Specify the date when each source was last searched or consulted.	Page 3 - Data search strategy and study selection
**Search strategy**	7	Present the full search strategies for all databases, registers and websites, including any filters and limits used.	Page 3 - Data search strategy and study selection
**Selection process**	8	Specify the methods used to decide whether a study met the inclusion criteria of the review, including how many reviewers screened each record and each report retrieved, whether they worked independently, and if applicable, details of automation tools used in the process.	Page 3 - Data extraction process and data items
**Data collection process**	9	Specify the methods used to collect data from reports, including how many reviewers collected data from each report, whether they worked independently, any processes for obtaining or confirming data from study investigators, and if applicable, details of automation tools used in the process.	Page 3 - Data extraction process and data items

Topic	No.	Item	Location where item is reported
**Data items**	10a	List and define all outcomes for which data were sought. Specify whether all results that were compatible with each outcome domain in each study were sought (e.g. for all measures, time points, analyses), and if not, the methods used to decide which results to collect.	Page 3 - Data extraction process and data items
	10b	List and define all other variables for which data were sought (e.g. participant and intervention characteristics, funding sources). Describe any assumptions made about any missing or unclear information.	Page 3 - Data extraction process and data items
**Study risk of bias assessment**	11	Specify the methods used to assess risk of bias in the included studies, including details of the tool(s) used, how many reviewers assessed each study and whether they worked independently, and if applicable, details of automation tools used in the process.	Page 3 - Risk of bias (RoB) assessment
**Effect measures**	12	Specify for each outcome the effect measure(s) (e.g. risk ratio, mean difference) used in the synthesis or presentation of results.	Page 3, 4 - Summary Measures and Synthesis of Results
**Synthesis methods**	13a	Describe the processes used to decide which studies were eligible for each synthesis (e.g. tabulating the study intervention characteristics and comparing against the planned groups for each synthesis (item 5)).	Not reported
	13b	Describe any methods required to prepare the data for presentation or synthesis, such as handling of missing summary statistics, or data conversions.	Page 4 - Summary Measures and Synthesis of Results
	13c	Describe any methods used to tabulate or visually display results of individual studies and syntheses.	Page 4 - Summary Measures and Synthesis of Results
	13d	Describe any methods used to synthesize results and provide a rationale for the choice(s). If meta-analysis was performed, describe the model(s), method(s) to identify the presence and extent of statistical heterogeneity, and software package(s) used.	Page 4 - Summary Measures and Synthesis of Results
	13e	Describe any methods used to explore possible causes of heterogeneity among study results (e.g. subgroup analysis, meta-regression).	Page 4 - Summary Measures and Synthesis of Results
	13f	Describe any sensitivity analyses conducted to assess robustness of the synthesized results.	Page 4 - Summary Measures and Synthesis of Results
**Reporting bias assessment**	14	Describe any methods used to assess risk of bias due to missing results in a synthesis (arising from reporting biases).	Page 3 - Risk of bias (RoB) assessment

Topic	No.	Item	Location where item is reported
**Certainty assessment**	15	Describe any methods used to assess certainty (or confidence) in the body of evidence for an outcome.	Not Reported
**RESULTS**			
**Study selection**	16a	Describe the results of the search and selection process, from the number of records identified in the search to the number of studies included in the review, ideally using a flow diagram.	Page 4, 5 - Study selection
	16b	Cite studies that might appear to meet the inclusion criteria, but which were excluded, and explain why they were excluded.	Table 2 Supplementary material
**Study characteristics**	17	Cite each included study and present its characteristics.	Page 5,6 - Studies characteristics
**Risk of bias in studies**	18	Present assessments of risk of bias for each included study.	Page 6 - Methodological Quality of the Included Studies
**Results of individual studies**	19	For all outcomes, present, for each study: (a) summary statistics for each group (where appropriate) and (b) an effect estimate and its precision (e.g. confidence/credible interval), ideally using structured tables or plots.	Page 7 to 10 - Data synthesis
**Results of syntheses**	20a	For each synthesis, briefly summarise the characteristics and risk of bias among contributing studies.	Page 7 to 10 - Data synthesis
	20b	Present results of all statistical syntheses conducted. If meta-analysis was done, present for each the summary estimate and its precision (e.g. confidence/credible interval) and measures of statistical heterogeneity. If comparing groups, describe the direction of the effect.	Page 7 to 10 - Data synthesis
	20c	Present results of all investigations of possible causes of heterogeneity among study results.	Page 7 to 10 - Data synthesis
	20d	Present results of all sensitivity analyses conducted to assess the robustness of the synthesized results.	Page 7 to 10 - Data synthesis
**Reporting biases**	21	Present assessments of risk of bias due to missing results (arising from reporting biases) for each synthesis assessed.	Not reported
**Certainty of evidence**	22	Present assessments of certainty (or confidence) in the body of evidence for each outcome assessed.	Page 7 to 10 - Data synthesis
**DISCUSSION**			

Topic	No.	Item	Location where item is reported
**Discussion**	23a	Provide a general interpretation of the results in the context of other evidence.	Page 11 - Implications for practice and research
	23b	Discuss any limitations of the evidence included in the review.	Page 11 - Strengths and limitations
	23c	Discuss any limitations of the review processes used.	Page 11 - Strengths and limitations
	23d	Discuss implications of the results for practice, policy, and future research.	Page 11 - Implications for practice and research
**OTHER INFORMATION**			
**Registration and protocol**	24a	Provide registration information for the review, including register name and registration number, or state that the review was not registered.	Page 2 - Protocol and registration
	24b	Indicate where the review protocol can be accessed, or state that a protocol was not prepared.	Page 2 - Protocol and registration
	24c	Describe and explain any amendments to information provided at registration or in the protocol.	Not reported
**Support**	25	Describe sources of financial or non-financial support for the review, and the role of the funders or sponsors in the review.	Page 12 - Funding
**Competing interests**	26	Declare any competing interests of review authors.	Page 12 - Conflicts of Interest
**Availability of data, code and other materials**	27	Report which of the following are publicly available and where they can be found: template data collection forms; data extracted from included studies; data used for all analyses; analytic code; any other materials used in the review.	Not reported

From: Page MJ, McKenzie JE, Bossuyt PM, Boutron I, Hoffmann TC, Mulrow CD, et al. The PRISMA 2020 statement: an updated guideline for reporting systematic reviews. MetaArXiv. 2020, September 14. DOI: 10.31222/osf.io/v7gm2. For more information, visit: www.prisma-statement.org [49].

Table S2. List of excluded studies with reasons.

	Reference	Year	Reason
[50]	Aggarwal A, Pai KM. Orofacial Mani- festations of Leukemic Children on Treatment: A Descriptive Study. Int J Clin Pediatr Dent 2018;11(3):193-198.	2018	Does not describe late side effects
[51]	Alberth M, Kovalecz G, Nemes J, Math J, Kiss C, Marton IJ. Oral Health of Long-Term Childhood Cancer Survivors. Pediatr Blood Cancer 2004;43:88–90	2004	Brief report
[52]	Ali MHM, Nurelhuda NM. Oral health status and its determinants in children with leukaemia at the Radiation and Isotope Center Khartoum, Khartoum State, Sudan. SUDANESE JOURNAL OF PAEDIATRICS 2019; Vol 19, Issue No. 2	2019	undergoing cancer therapy
[53]	Azher U, Shiggaon N. Oral health status of children with acute lymphoblastic leukemia undergoing chemotherapy. Indian J Dent Res [serial online] 2013 [cited 2022 Apr 29];24:523. Available from: https://www.ijdr.in/text.asp?2013/24/4/523/118371	2022	evaluate the oral health status of children with acute lymphoblastic leukemia undergoing chemotherapy
[54]	Berger Velten D, Zandonade E, Monteiro de Barros Miotto MH. Prevalence of oral manifestations in children and adolescents with cancer submitted to chemotherapy. BMC Oral Health. 2016 Oct 3;16(1):107. doi: 10.1186/s12903-016-0300-2. PMID: 27716167; PMCID: PMC5047274.	2016	undergoing cancer therapy
[55]	Childers NK, Stinnett EA, Wheeler P, Wright JT, Castleberry RP, Dasanayake AP, Ala B, Ala D, Hill C. Oral complications in children with cancer. Oral Surg Oral Med Oral Pathol, January 1993	1993	Oral complications in children with cancer undergoing cancer therapy
[56]	Costa RC, Bezerra PMM, Damascena LCL, Ribeiro IL, Bonan PRF, Sousa SA, Almeida LFD, Valença AMG. Impact of Saliva and Cariogenic Microbiota on the Chemotherapy-Induced Oral Mucositis in Oncopediatric Patients: A Preliminary Longitudinal Study. International Journal of Dentistry Volume 2020, Article ID 1243953, https://doi.org/10.1155/2020/1243953	2020	Impact of Saliva and Cariogenic Microbiota in children with cancer undergoing cancer therapy
[57]	Curtis A. Childhood leukemias: initial oral manifestations. JADA, Vol. 83, July 1971	1971	Initial oral manifestations at the time of diagnose and undergoing cancer therapy

[58]	El-Housseiny AA, Saleh SM, El-Masry AA, Allam AA. Assessment of Oral Complications in Children Receiving Chemotherapy. The Journal of Pediatric Dentistry Volume 31, Number 4/2007	2004	undergoing cancer therapy
[59]	Fayle SA, Curzon MEJ. Oral Complications in pediatric oncology patients. Pediatric Dentistry: September/October, 1991-Volume 12, Number 5	1991	undergoing cancer therapy
[60]	Gandhi K, Datta G, Ahuja S, Saxena T, Datta AG. Prevalence of Oral Complications occurring in a Population of Pediatric Cancer Patients receiving Chemotherapy. Int Int J Clin Pediatr Dent 2017;10(2):166-171.	2017	undergoing cancer therapy
[61]	González-Gravina H, González-de Morán E, Zambrano O, Lozano-Chourio M, Rodríguez-de Valero S, Robertis S, Mesa L. Oral Candidiasis in children and adolescents with cancer. Identification of Candida spp. Med Oral Patol Oral Cir Bucal. 2007 Oct 1;12(6):E419-23.	2007	undergoing cancer therapy
[62]	Gupta A, Marwaha M, Bansal K, Sachdeva A, Gupta A. Dental Awareness among Parents and Oral Health of Paediatric Cancer Patients Receiving Chemotherapy. Journal of Clinical and Diagnostic Research. 2016 May, Vol-10(5)	2016	undergoing cancer therapy
[63]	Hedge AM, Joshi S, Rai K, Shetty S. Evaluation of Oral Hygiene Status, Salivary Characteristics and Dental Caries Experience in Acute Lymphoblastic Leukemic (ALL) Children. The Journal of Clinical Pediatric Dentistry Volume 35, Number 3/2011	2011	Children diagnosed with Acute Lymphoblastic Leukemia and undergoing cancer therapy
[64]	Hovi L, Saarinen UM, Donner U, Lindquist C. Opportunistic Osteomyelitis in the jaws of Children on Immunosuppressive Chemotherapy. Journal of Paediatric Hematology/Oncology 18(1):90-94, 1996	1996	undergoing cancer therapy
[65]	Kaste SC, Hopkins KP, Jenkins JJ. Abnormal odontogenesis in Children treated with radiation and Chemotherapy: Imaging Findings. American Journal of Roentgenology – July 1994	1994	Review
[66]	Kowlessar A, Naidu R, Ramroop V, Nurse J, Dookie K, Bodkyn C, Lalchandani S. Oral health among children attending an oncology clinic in Trinidad. Clin Exp Dent Res. 2019;1–5.	2019	undergoing cancer therapy
[67]	Kung AY, Zhang S, Zheng LW, Wong GH, Chu CH. Oral health status of chinese paediatric and adolescent oncology patients with chemotherapy in Hong Kong: a	2015	undergoing cancer therapy

	pilot study. Open Dent J. 2015 Jan 30;9:21-30. doi: 10.2174/1874210601509010021. PMID: 25674168; PMCID: PMC4319200.		
[68]	Levy-Polack MP, Sebelli P, Polak NL. Incidence of oral complications and application of a preventive protocol in children with acute leukemia. SCD Special Care in Dentistry, 1998, V18,N5	1998	undergoing cancer therapy
[69]	Lula ECO, Lula CEO, Alves CMC, Lopes FF, Pereira ALA. Chemotherapy-induced oral complications in leukemic patients. International Journal of Pediatric Otorhinolaryngology (2007) 71, 1681—1685	2007	undergoing cancer therapy
[70]	Marangoni-Lopes L, Rodrigues LP, Mendonça RH, Nobre-dos-Santos M. Radiotherapy changes salivary properties and impacts quality of life of children with Hodgkin disease. Archives of Oral Biology 72 (2016) 99–105	2016	undergoing cancer therapy
[71]	Mathur VP, Dhillon JK, Kalra G. Oral Health in Children with Leukemia. Indian J Palliat Care. 2012 Jan-Apr; 18(1): 12–18.	2012	Complications of Leukemia
[72]	Morais EF, Lira JAS, Macedo RAP, Santos KS, Elias CTV, Arruda-Morais MLS. Oral manifestations resulting from chemotherapy in children with acute lymphoblastic leukemia. Braz J Otorhinolaryngol. 2014;80:78-85.	2014	undergoing cancer therapy
[73]	Mougeot JC, Stevens CB, Almon KG, Paster BJ, Lalla RV, Brennan MT, Mougeot FB. Caries-associated oral microbiome in head and neck cancer radiation patients: a longitudinal study. J Oral Microbiol. 2019 Mar 8;11(1):1586421. doi: 10.1080/20002297.2019.1586421. PMID: 30891159; PMCID: PMC6419625.	2019	undergoing cancer therapy
[74]	Nasim VS, Shetty YR, Hedge AM. Dental Health Status in Children with Acute Lymphoblastic Leukemia. The Journal of Pediatric Dentistry Volume 31, Number 3/2007	2007	Complications of Leukemia and undergoing cancer therapy
[75]	Neto AEM, Westphalen FH. Analysis of oral complications related to cancer therapy. Arch Oral Res. 2013 May/Aug.;9(2)159-164	2013	undergoing cancer therapy
[76]	Nikoui M, Lalonde B. Manifestations bucco-dentaires de la leucémie chez l'enfant [Oro-dental manifestations of leukemia in children]. J Can Dent Assoc. 1996 May;62(5):443-6, 449-50. French. PMID: 8640581.	1996	Review

[77]	O'Sullivan EA, Duggal MS, Bailey CC. Changes in the oral health of children during treatment for acute lymphoblastic leukaemia. International Journal of Paediatric Dentistry 1994; 4:31-34	1994	undergoing cancer therapy
[78]	Olszewska K, Mielnik-Blaszczar. An Assessment of the Number of Cariogenic Bacteria in the Saliva of Children with Chemotherapy-Induced Neutropenia. Adv Clin Exp Med 2016, 25, 1, 11–19. DOI: 10.17219/acem/28998	2016	undergoing cancer therapy
[79]	Orback R, Orbak Z. Oral condition of patients with leukemia and lymphoma J. Nihon Univ. Sch. Dent., Vol. 39, No. 2, 67-70, 1997	1997	Complications of Leukemia and lymphoma
[80]	Ou-Yang L, Chanh P, Tsai A, Jaing T, Lin S. Salivary Microbial Counts and Buffer Capacity in Children with Acute lymphoblastic leukemia. Pediatric Dentistry 2010, V 32;No3	2010	undergoing cancer therapy
[81]	Parra JJ, Alvarado MC, Monsalve P, Costa ALF, Montesinos GA, Parra PA. Oral health in children with acute lymphoblastic leukaemia: before and after chemotherapy treatment. European Archives of Paediatric Dentistry https://doi.org/10.1007/s40368-019-00454-4		undergoing cancer therapy
[82]	Ponce-Torres E, Ruíz-Rodríguez MS, Alejo-González F, Hernández-Sierra JF, Pozoz-Guillén AJ. Oral Manifestations in Pediatric Patients Receiving Chemotherapy for Acute Lymphoblastic Leukemia. J Clin Pediatr Dent 34(3): 275–280, 2010	2010	undergoing cancer therapy
[83]	Ribeiro ILA, Silva SM, Limeira RRT, Bonan PRF, Valença AMG, Lima Neto EA, Castro RD. Differences between the oral changes presented by patients with solid and hematologic tumors during the chemotherapeutic treatment. J Appl Oral Sci. 2020;28:e20190020	2020	undergoing cancer therapy
[84]	Ritwik P. Dental Care for Patients With Childhood Cancers. Ochsner J. 2018 Winter; 18(4): 351–357.	2018	Review
[85]	Ryan ME, Hopkins K, Wilbur RB. Acute necrotizing Ulcerative Gengivitis in Children with Cancer. Am J Dis Child – Vol 137, June 1983.	1983	undergoing cancer therapy
[86]	Sahai P, Mohanti BK, Sharma A, Thakar A, Bhasker S, Kakkar A, Sharma MC, Upadhyay AD. Clinical outcome and morbidity in pediatric patients with nasopharyngeal cancer treated with chemoradiotherapy. PediatrBloodCancer2016;00:1–8	2016	Oral or dental status not addressed

[87]	Sepúlveda-Tebache E, Brethauer-Meier U, Jiménez-Moraga M, Mora- les-Figueroa R, Rojas-Castro J, Le Fort-Canales P. Detección del virus herpes simple en lesiones de la mucosa oral en pacientes con terapia oncológica. Med Oral 2003;8:329-33	2003	undergoing cancer therapy
[88]	Tao CJ, Liu X, Tang LL, Mao YP, Chen L, Li WF, Yu XL, Liu LZ, Zhang R, Lin AH, Ma J, Sun Y. Long-term outcome and late toxicities of simultaneous integrated boost-intensity modulated radiotherapy in pediatric and adolescent nasopharyngeal carcinoma. Chin J Cancer. 2013 Oct;32(10):525-32. doi: 10.5732/cjc.013.10124. Epub 2013 Sep 10. PMID: 24016394; PMCID: PMC3845542.	2013	Oral or dental status not addressed
[54]	Velten DB, Zandonade E, Miotto MHMB. Prevalence of oral manifestations in children and adolescents with cancer submitted to chemotherapy. BMC Oral Health (2016) 16:107	2016	evaluate changes in oral lesions in children and adolescents with cancer undergoing chemotherapy
[89]	Velten DB, Zandonade E, Miotto MHMB. Prevalence of oral manifestations in children and adolescents with cancer submitted to chemotherapy. BMC Oral Health (2017) 17:49	2017	undergoing cancer therapy
[90]	Visnapuu V, Peltonen S, Alivuotila L, Happonen R-P, Peltonen J. Craniofacial and oral alterations in patients with Neurofibromatosis 1. Orphanet Journal of Rare Diseases (2018) 13:131	2018	Craniofacial and oral alterations at the time of diagnose
[91]	Wang Y, Zeng X, Yang X, Que J, Du Q, Zhang Q, Zou J. Oral Health, caries risk profiles, and oral microbiome of pediatric patients with Leukemia submitted to chemotherapy. BioMed research International Jan 2021;	2021	undergoing cancer therapy
[92]	White GE, Cambridge M. Oral manifestations of leukemia in children. Oral Surg March 1970; Vol.29:No3	1970	oral manifestation of lekemia

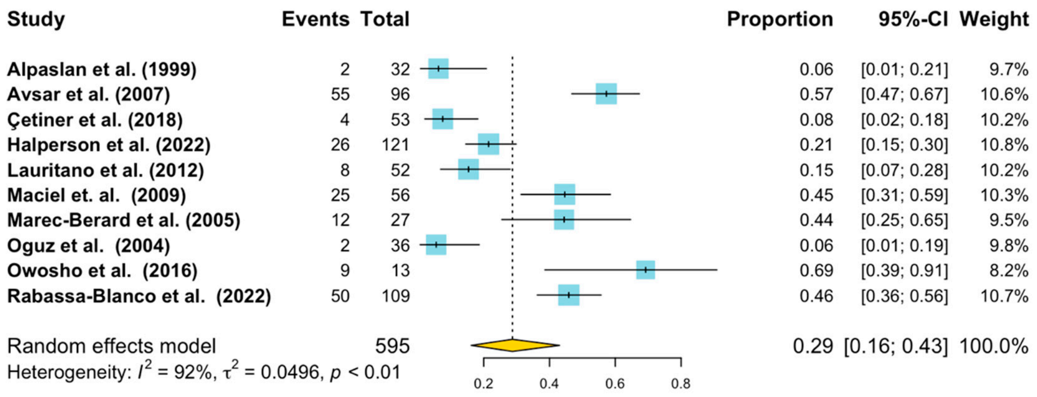

Supplementary file 3. Forest plot of prevalence of root development alteration in survival pediatric cancer in comparison to those without root alterations, with mean effect size estimates and 95% confidence intervals. The size of squares reflects sample size, while continuous horizontal lines and the width of diamonds indicate the 95% confidence interval. The diamond and vertical dotted line represent the overall pooled estimate of root development alteration prevalence.

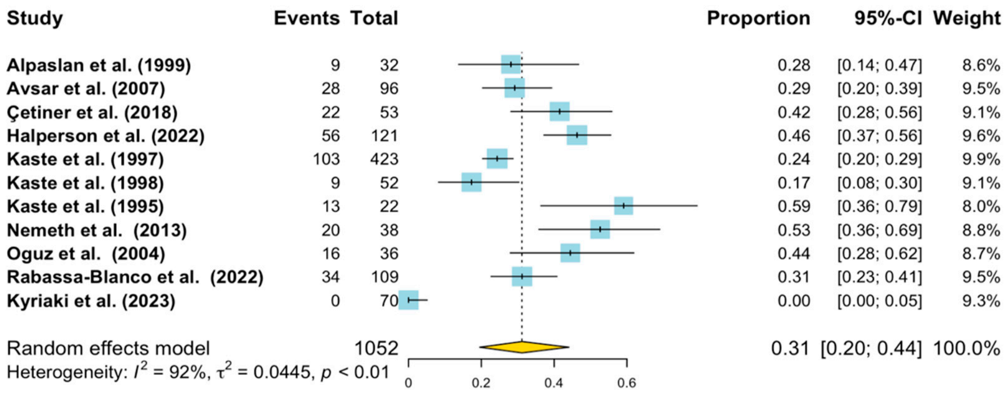

Supplementary file 4. Forest plots of prevalence of crown-root malformation.

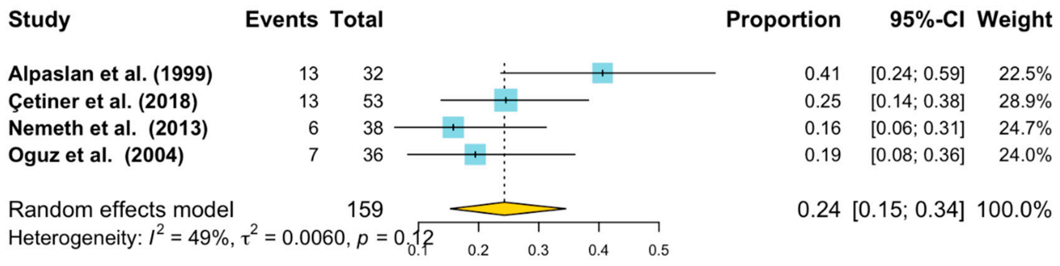

Supplementary file 5. Forest plots of prevalence of unerupted teeth.

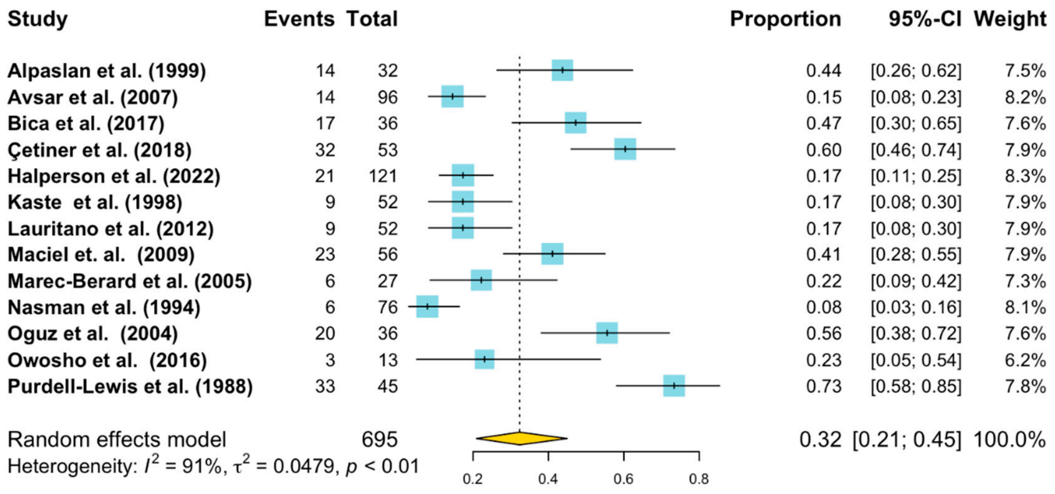

Supplementary file 6. Forest plots of prevalence of enamel hypoplasia.

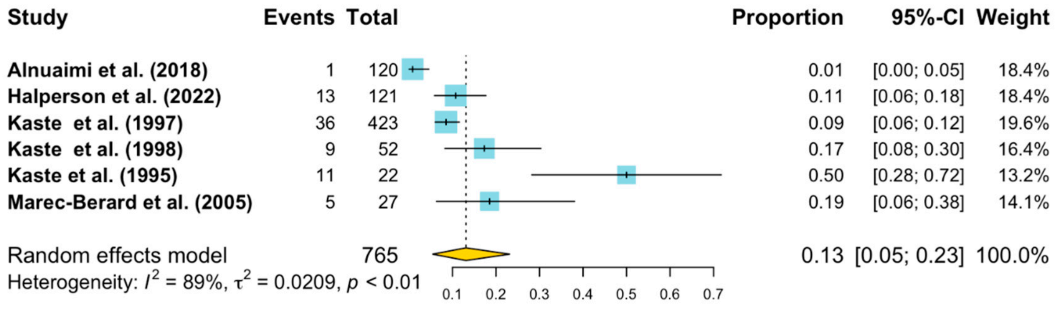

Supplementary file 7. Forest plots of prevalence of hypodontia.

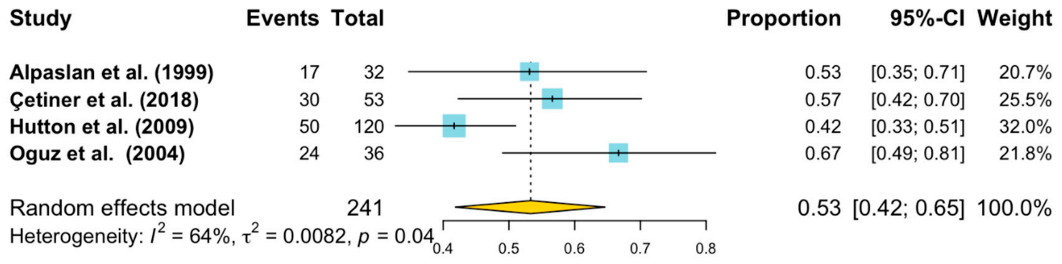

Supplementary file 8. Forest plots of prevalence of discoloration.

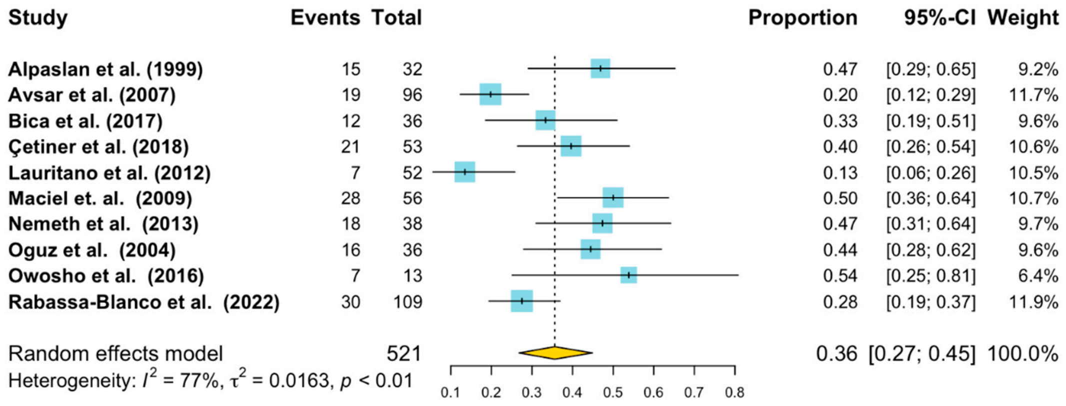

Supplementary file 9. Forest plots of prevalence of agenesis.

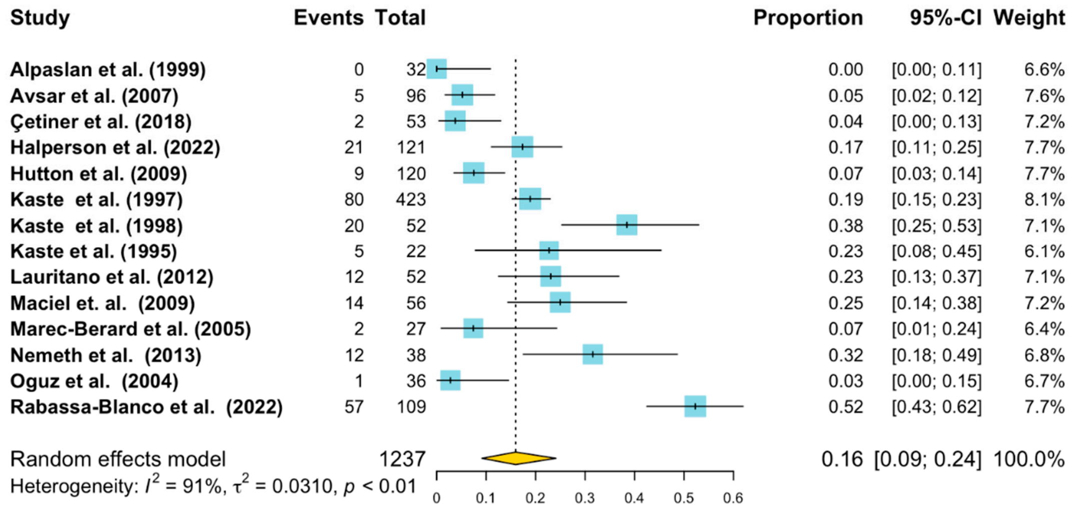

Supplementary file 10. Forest plots of prevalence of microdontia.

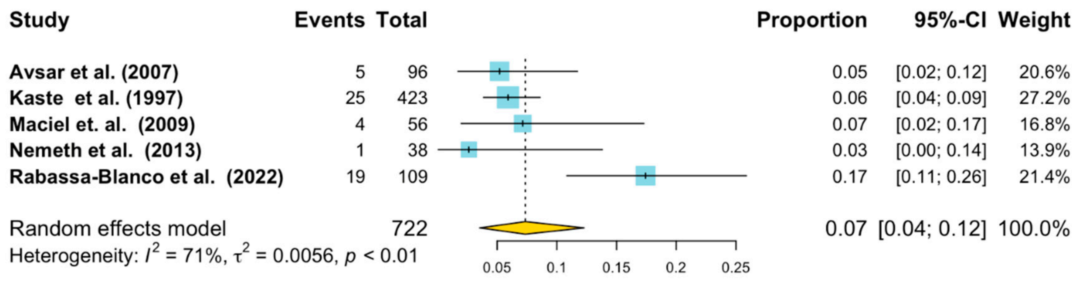

Supplementary file 11. Forest plots of prevalence of macrodontia.

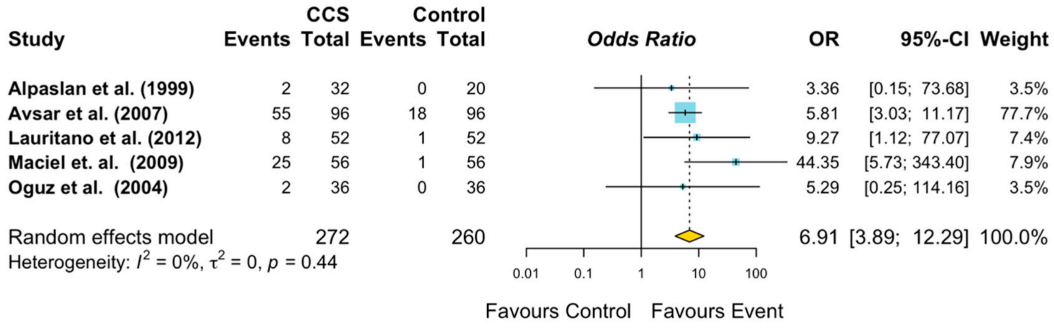

Supplementary file 12. Forest plots of odds ratio of root development alteration.

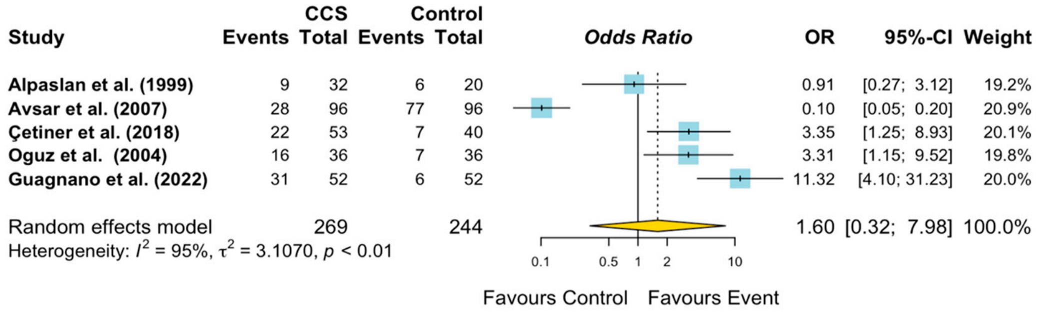

Supplementary file 13. Forest plots of odds ratio of crown.

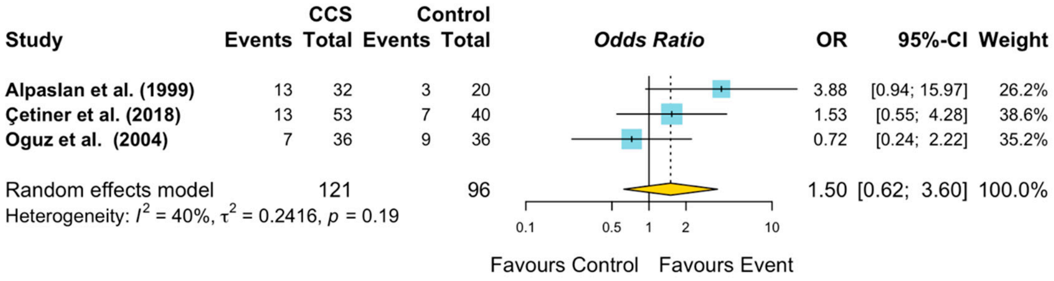

Supplementary file 14. Forest plots of odds ratio of unerupted teeth.

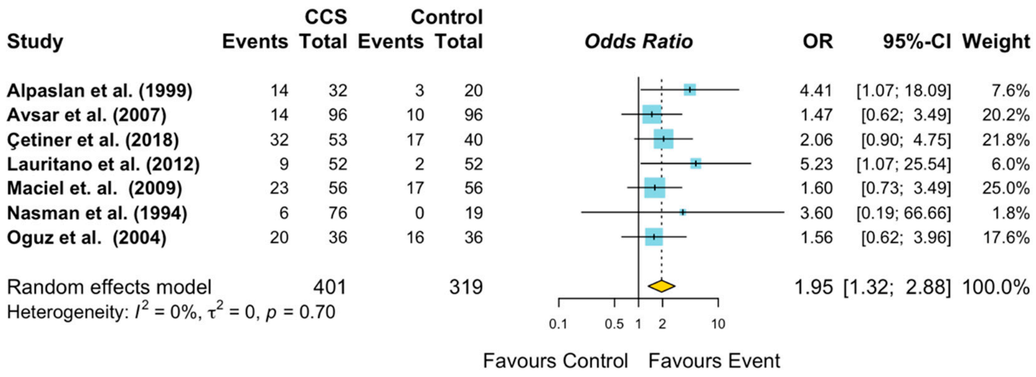

Supplementary file 15. Forest plots of odds ratio of enamel hypoplasia.

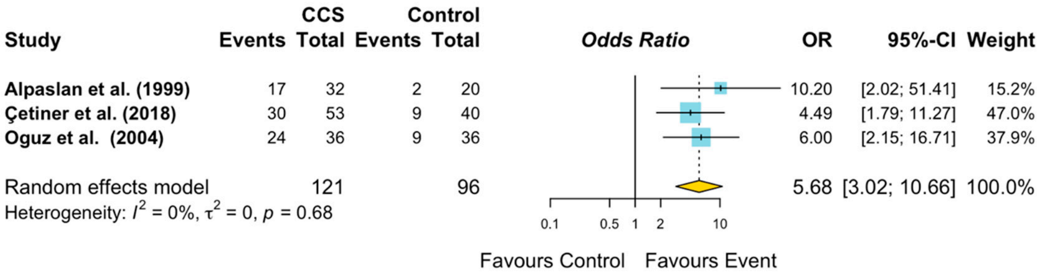

Supplementary file 16. Forest plots of odds ratio of discoloration.

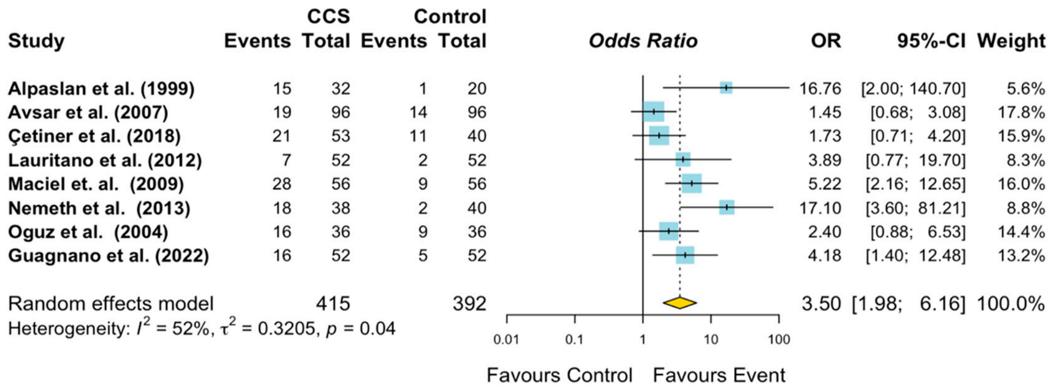

Supplementary file 17. Forest plots of odds ratio of agenesis.

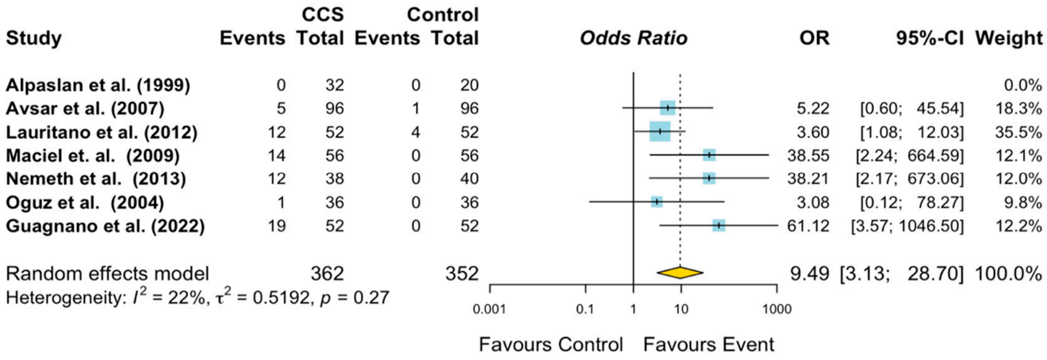

Supplementary file 18. Forest plots of odds ratio of microdontia.

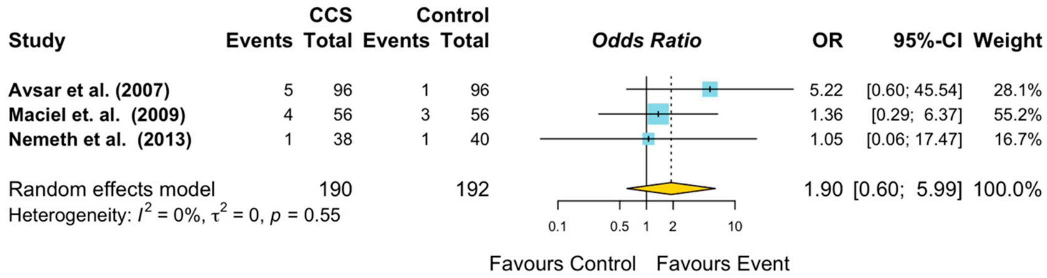

Supplementary file 19. Forest plots of odds ratio of macrodontia.
